# Antipsychotic Drugs: The Antithesis to Neurorehabilitation in Models of Pre-Clinical Traumatic Brain Injury

**DOI:** 10.1089/neur.2023.0082

**Published:** 2023-10-31

**Authors:** Nicholas S. Race, Eleni H. Moschonas, Jeffrey P. Cheng, Corina O. Bondi, Anthony E. Kline

**Affiliations:** ^1^ Physical Medicine & Rehabilitation, University of Pittsburgh, Pittsburgh, Pennsylvania, USA.; ^2^ Safar Center for Resuscitation Research, University of Pittsburgh, Pittsburgh, Pennsylvania, USA.; ^3^ Center for Neuroscience, University of Pittsburgh, Pittsburgh, Pennsylvania, USA.; ^4^ Neurobiology, University of Pittsburgh, Pittsburgh, Pennsylvania, USA.; ^5^ Center for the Neural Basis of Cognition, University of Pittsburgh, Pittsburgh, Pennsylvania, USA.; ^6^ Psychology, University of Pittsburgh, Pittsburgh, Pennsylvania, USA.; ^7^ Critical Care Medicine, University of Pittsburgh, Pittsburgh, Pennsylvania, USA.; ^8^ Association of Academic Physiatrists Rehabilitation Medicine Scientist Training Program, Owings Mills, Maryland, USA.

**Keywords:** antipsychotic drugs, behavior, cognition, controlled cortical impact, Morris water maze, recovery, traumatic brain injury

## Abstract

Sixty-nine million traumatic brain injuries (TBIs) are reported worldwide each year, and, of those, close to 3 million occur in the United States. In addition to neurobehavioral and cognitive deficits, TBI induces other maladaptive behaviors, such as agitation and aggression, which must be managed for safe, accurate assessment and effective treatment of the patient. The use of antipsychotic drugs (APDs) in TBI is supported by some expert guidelines, which suggests that they are an important part of the pharmacological armamentarium to be used in the management of agitation. Despite the advantages of APDs after TBI, there are significant disadvantages that may not be fully appreciated clinically during decision making because of the lack of a readily available updated compendium. Hence, the aim of this review is to integrate the existing findings and present the current state of APD use in pre-clinical models of TBI. The studies discussed were identified through PubMed and the University of Pittsburgh Library System search strategies and reveal that APDs, particularly those with dopamine_2_ (D_2_) receptor antagonism, generally impair the recovery process in rodents of both sexes and, in some instances, attenuate the potential benefits of neurorehabilitation. We believe that the compilation of findings represented by this exhaustive review of pre-clinical TBI + APD models can serve as a convenient source for guiding informed decisions by critical care clinicians and physiatrists contemplating APD use for patients exhibiting agitation.

## Introduction

Recent estimates of world-wide traumatic brain injury (TBI) report 69 million cases annually,^1^ of which nearly 3 million occur in the United States.^2–4^ In addition to motor and cognitive deficits, which are prevalent and often persistent,^5–11^ as well as numerous secondary pathophysiological effects,^12–16^ TBI induces other maladaptive behaviors^17^ like agitation and aggression.

Agitation post-TBI is distressing for patients, families, and staff and is associated with worse outcomes.^18–23^ Agitation is a loosely defined term describing a collection of behaviors, including restlessness, distractibility, impulsivity, anger, aggression, and violent verbal and/or physical outbursts.^24^ An agitated patient may exhibit one, some, or all of these behaviors, generating a range of neurobehavioral phenotypes as diverse as the nearly unlimited combinations of injury severities leading to them. Defining which behaviors constitute agitation varies by clinician.^24^ In attempts to reduce subjectivity in the interpretation of agitation and its severity, scoring systems such as the Agitated Behavior Scale (ABS) have been developed.^25,26^ A 2021 study found that 75% of >300 surveyed clinicians use such scoring tools^24^; however, scoring utility has not been formally validated outside the original studies, and scoring cutoffs warranting intervention are not universally agreed upon.^27^ In this vein, some studies note that up to one third of TBI patients are prescribed antipsychotic drugs (APDs) despite lacking clinically significant agitation by ABS scores.^27,28^ Issues with the consistent definition of, and characterization methods for, agitation are compounded by the perceived inadequacy of tools for and training in agitation management by the majority of clinicians.^24^

Research into appropriate interventions for agitation after TBI is also limited.^17,29^ In a systematic review and meta-analysis on drugs for treating agitation after TBI, Williamson and colleagues^30^ concluded that “although agitation is frequent following TBI and pharmacological agents are often used, there is no consensus on the most efficacious and safest strategy to treat these complications.” In a systematic review, Nash and colleagues (2019)^31^ stated that “the pharmacological management of agitated patients immediately following a TBI is still an area of much-needed research, as there is limited data-driven guidance in the literature.” Given the paucity of randomized trials on TBI + agitation, pharmacological treatment options are inherited from other diagnostic entities with similar symptomatology (e.g., schizophrenia and dementia) and applied to TBI patients without a full understanding of the specific risks unique to TBI.^32^ Neuroleptics are one such class of drugs.

APDs are used with great frequency after TBI.^29–38^ The rationale for using APDs post-TBI is that they reduce agitation when the risk of injury to self or others is imminent, and when the condition prevents the patient from receiving appropriate acute care or rehabilitation. A review of the literature for existing APDs of choice for combatting agitation are numerous for schizophrenia but limited to case reports for TBI.^38–41^ In addition to haloperidol (HAL) and risperidone (RISP), other first- and second-generation APDs for TBI include clozapine, ziprasidone, olanzapine, quetiapine, and aripiprazole (ARIP) as first choices.^42–44^ APDs are “dirty” drugs and act on several receptors (e.g., serotonin [5-HT], 5-HT_2A_, 5-HT_2C_, 5-HT_6_, and 5-HT_7_; dopamine, D_2_, and D_4_; muscarinic acetylcholine receptor M1, histamine H1, and alpha 1-adrenergic). Additional APDs are available but used infrequently,^39,41,42^ as are non-APD drugs like beta-blockers (e.g., propranolol, pindolol), benzodiazepines, buspirone, and amantadine.^39^ Regardless of the options available, HAL and RISP continue to be used given that they work quickly.^45^

In communications with critical care and rehabilitation physicians, it is acknowledged that although aware of the negative effects on motor and cognition, the primary goal is to combat agitation; and because HAL produces rapid effects (i.e., sedation), it is often a go-to drug. A Cochran review found that more injections of ARIP, a second-generation APD, were needed to reduce the same level of agitation as a single injection of HAL.^46^ There is currently some movement to refrain from using HAL,^42,45^ but it currently remains among the most frequently used medications for agitation management. The aim of this review on APDs after pre-clinical TBI is to integrate the findings and highlight the consequences of APDs on behavioral outcomes. We believe that the compilation of findings can serve as a handy source for critical care clinicians and physiatrists contemplating APD use for their agitated patients.

## Methods

### Article search

The specific key-term phrase “traumatic brain injury AND rodents AND antipsychotic drugs” yielded 32 and 29 TBI articles by PubMed and the University of Pittsburgh Library System, respectively. After reviewing the articles to ensure that they fit the criteria of TBI (i.e., cortical injury by controlled pneumatic impact, weight drop, or fluid pulse) in rodents undergoing behavioral outcome assessments after the administration of APDs, 12 articles were common to both search strategies. PubMed and a review of the bibliographies yielded three other potential articles, but, upon further review, it was noted that the APD (trifluoperazine) is no longer commonly used in clinical practice and thus was omitted as was a study where a pharmacological manipulation was conducted before TBI and APD administration, which is inconsistent with the selected articles. Articles reporting brain injury by aspiration or electrolytic lesions were also not included. In addition to the key terms, other inclusion criteria were the use of vehicle-treated controls, randomization, and blinding. All 13 studies that fit the criteria are described in the text and summarized in Table 1.

**Table 1.** Summary of Neurobehavioral and Cognitive Outcomes Induced by Antipsychotic Drugs After Pre-Clinical Traumatic Brain Injury Presented in the Order Discussed in Main Text Body

***Model***	***Treatments***	***Assessments***	***Results***	***Reference***
CCI, adult male rats	• RISP (0.045, 0.45, and 4.5 mg/kg, i.p.), HAL (0.5 mg/kg, i.p.), or VEH (1 mL/kg, i.p.) • Phase 1: single administration 24 h after CCI injury or sham surgery • Phase 2: once-daily, post-operative days 19–23 • Phase 3: no drug administrations	• Beam balance: days 1–5, 20–23, and 26 for phases 1–3, respectively • Beam walk: days 1–5, 20–23, and 26 for phases 1–3, respectively • Spatial learning: days 14–18, 20–23, and 26 for phases 1–3, respectively	• Phase 1: no group differences in motor or cognition • Phase 2: RISP (4.5 mg/kg) and HAL did not differ from each other, but impaired motor and cognition vs. VEH and lower doses of RISP. • Phase 3: RISP (4.5 mg/kg) and HAL did not differ from each other, but impaired cognition vs. VEH and lower doses of RISP.	Kline et al., 2007^50^
Weight-drop mTBI, adult male mice	• HAL (0.3, 1.0, and 3.0 mg/kg, i.p.), or VEH • Single administration 15 min after TBI	• Water-finding task: acquisition, retention, and retest on days 7, 8, and 9, respectively.	• HAL (all three doses) improved acquisition and retention vs. VEH.	Tang et al.,1997^47^
CCI, adult male rats	• HAL (0.5 mg/kg, i.p.), RISP (0.045, 0.45, and 4.5 mg/kg, i.p.), or VEH (1 mL/kg, i.p.) • Treatments began 24 h after TBI or sham surgery (1 h before daily behaviors) and continued once-daily for 19 days.	• Beam walk: days 1–5 • Acquisition of spatial learning: days 14–18 • Swim speed: day 19	• RISP (all doses) and HAL hindered the ability to recover TBI-induced motor and cognitive deficits vs. VEH. • RISP and HAL impaired motor and cognitive ability in sham controls. • RISP and HAL slowed swim speed in TBI and sham controls.	Kline et al., 2008^51^
CCI, adult male rats	• HAL (0.5 mg/kg, i.p.), RISP (0.45 mg/kg, i.p.), or VEH (1 mL/kg, i.p.) • Treatments began 24 h after TBI or sham surgery (after daily behaviors) and continued once-daily for 19 days.	• Beam walk: days 1–5 • Acquisition of spatial learning: days 14–18 • Swim speed: day 19 • Hippocampal CA1/CA2 neurons: day 21 Cortical lesion volume: day 21	• RISP and HAL delayed motor and inhibited cognitive recovery vs. VEH. • No differences among the groups, regardless of treatment in hippocampal CA1 and CA3 survival or cortical lesion volume	Hoffman et al., 2008^52^
FPI, adult male rats	• HAL (0.03, 0.1, and 0.3 mg/kg; i.p.), olanzapine (0.3, 1.0, and 3.0 mg/kg; i.p.), and VEH (1 mL/kg, i.p.) • Treatments began 24 h after TBI or sham surgery and continued once-daily for 15 days.	• Beam balance, beam walk: days 1–5 • Acquisition of spatial learning: days 11–15	• Neither HAL nor olanzapine affected motor vs. VEH. • HAL (0.3 mg/kg) impaded the acquisition of spatial learning vs. VEH and HAL (0.1 mg/kg). • Olanzapine did not affect spatial learning vs. VEH.	Wilson et al., 2003^48^
CCI, adult male and female rats	• HAL (0.5 mg/kg, i.p.) or VEH (1 mL/kg, i.p.) • Treatments began 24 h after CCI or sham surgery and continued once-daily for 19 days.	• Beam-balance, beam-walk time, and beam-walk score: days 1–5 • Acquisition of spatial learning: days 14–18 • Cortical lesion volume: day 21	• HAL inhibited motor recovery in male rats. No HAL-induced inhibition observed in females • HAL inhibited the acquisition of spatial learning in both sexes equally. • HAL did not affect cortical lesion volume vs. VEH in males. • Females had smaller cortical lesions regardless of treatment.	Free et al., 2017^49^
CCI, adult male rats	• HAL (0.5 mg/kg, i.p.), RISP (0.45 mg/kg, i.p.), BRO (5.0 mg/kg, i.p.), or VEH (1 mL/kg, i.p.) • Treatments began 24 h after TBI or sham surgery and once-daily for 19 days.	• Beam balance and beam walk: days 1–5, 48, and 108 • Acquisition of spatial learning: days 14–18, 48, and 108 • Memory retention: days 19, 49, and 109 • Cortical lesion volume: day 109	• No motor differences revealed among the TBI groups at any time point • HAL impeded cognitive recovery during treatment and at 1 and 3 months vs. VEH. • RISP impeded cognitive recovery during treatment and at 1 month vs. VEH. • BRO enhanced spatial learning and memory retention vs. all other TBI groups during treatment and at 1 and 3 months. • BRO reduced cortical lesion volume vs. HAL, RISP, and VEH.	Phelps et al., 2015^53^
CCI, adult male rats	• ARIP (0.1, 1.0 mg/kg; i.p.), VEH (1 mL/kg, i.p.) • Treatments began 24 h after TBI or sham surgery and continued once-daily for 19 days.	• Beam-balance, beam-walk time: days 1–5 • Acquisition of spatial learning: days 14–18 • Memory retention: day 19 • Cortical lesion volume and hippocampal cell survival: day 19	• ARIP (1.0 mg/kg) facilitated beam-balance vs. VEH. • ARIP (0.1 mg/kg) improved spatial learning and memory retention vs. VEH. • Both ARIP groups decreased cortical lesion volume and increased hippocampal CA3 cell survival vs. VEH.	Phelps et al., 2017^54^
CCI, adult male rats	• HAL (0.5 mg/kg, i.p.) or VEH (1 mL/kg, i.p.) • Treatments began 24 h after CCI and continued either once-daily or once every other day for 19 days. • EE was initiated immediately after CCI and provided continuously for 19 days.	• Beam-balance, beam-walk time, and beam-walk score: days 1–5 • Acquisition of spatial learning: days 14–18 • Memory retention: day 19 • Cortical lesion volume: day 21	• Daily HAL in STD conditions impaired motor and cognitive recovery vs. VEH. • Daily HAL in EE attenuated the benefits of EE in cognitive recovery. • Intermittent HAL in EE did not disrupt spontaneous motor or cognitive recovery and did not differ from EE in memory retention or cortical lesion volume.	Bao et al. 2019^55^
CCI, adult male rats	• RISP (0.45 mg/kg, i.p.) or VEH (1.0 mL/kg, i.p.) • Treatments began 24 h after CCI and continued either once-daily for 19 days or one or three times per week until the culmination of behavioral assessments.	• Beam-walk time and beam-walk score: days 1–5 • Acquisition of spatial learning: days 14–18 • Memory retention: day 19	• RISP (seven times per week) exhibited greater motor and cognitive impairment compared to the groups receiving RISP one or three times per week, or VEH. • RISP (one or three times per week) did not differ from VEH. • No differences observed among TBI groups in memory retention	Carlson et al., 2018^56^
CCI, adult male rats	• HAL (0.5 mg/kg, i.p.), QUE (10 mg/kg, i.p.), or VEH (1 mL/kg, i.p.) • Treatments began 24 h after TBI or sham surgery and continued either once-daily (continuous) or once every other day (intermittent) for 19 days.	• Beam-balance and beam-walk time: days 1–5 • Acquisition of spatial learning: days 14–18 • Memory retention: day 19	• Daily HAL exhibited a difference on day 5 of beam walking vs. all other TBI groups. • Daily HAL significantly impaired spatial learning relative to all other TBI groups, which did not differ from one another. • Intermittent HAL did not impair motor and cognitive deficits. • Neither continuous nor intermittent QUE improved nor exacerbated TBI-induced motor and cognitive deficits.	Weeks et al., 2016^57^
CCI, adult male rats	• HAL (0.5 mg/kg; i.p.), VEH (1 mL/kg, i.p.) • Treatments began 24 h after TBI or sham surgery and continued once-daily for 19 days. • Treatments combined with STD or EE	• Beam balance, beam walk: days 1–5 • Acquisition of spatial learning: days 14–18 • Memory retention: day 19 • Cortical lesion volume: day 19	• EE VEH performed better than all groups on beam balance. • EE VEH and EE HAL performed better on beam-walk time and spatial learning. • EE VEH improved memory, but EE HAL did not. • Cortical lesion volume did not differ among the groups. • The therapeutic effects of EE were greatly reduced by concomitant administration of HAL.	Folweiler et al., 2017^58^
CCI, adult male rats	• ARIP (0.1 mg/kg; i.p.), VEH (1 mL/kg, i.p.) • Treatments began 24 h after TBI or sham surgery and continued once-daily for 19 days. • Treatments combined with STD or EE	• Beam-walk time, beam-walk score: days 1–5 • Acquisition of spatial learning: days 14–18 • Memory retention: day 19 • Cortical lesion volume: day 21	• ARIP-treated STD group performed better than VEH-treated STD on beam-walk score and spatial learning. • Both EE groups (ARIP and VEH) improved motor and cognitive outcomes vs. VEH STD and did not differ from one another. • Cortical lesion volume was decreased in all treatment groups vs. VEH STD.	Besegar et al., 2019^59^

ARIP, aripiprazole; CCI, controlled cortical impact; EE, environmental enrichment; FPI, fluid percussion injury; HAL, haloperidol; i.p., intraperitoneally; mTBI, mild traumatic brain injury; QUE, quetiapine; RISP, risperidone; STD, standard housing; TBI, traumatic brain injury; VEH, vehicle.

### Procedures (traumatic brain injury and behavioral assessments)

To reduce repetition in the description of TBI and behavioral assessments for each study discussed utilizing the controlled cortical impact (CCI) injury model, which comprise 84.6% of the reviewed articles, a brief mention of the procedures is provided. Procedures for the other two studies^47,48^ will be revealed during the Discussion because they are few and varied.

Briefly, subjects were overwhelmingly adult male rats except for a study by Free and colleagues (2017),^49^ which included females. A CCI injury of moderate severity (2.8-mm tissue deformation) or sham surgery was performed under a surgical level of anesthesia. Drugs were administered intraperitoneally (i.p.). Motor function was assessed using well-established beam-balance/walk tests that consist of training rats to balance for 60 sec and traversing an elevated narrow beam. Performance was measured by time on the beam before falling and time to traverse the beam. Spatial learning was assessed using a Morris water maze task. Acquisition of spatial learning began on post-operative day 14 and consisted of providing a block of four daily trials for 5 consecutive days (14–18) to locate the escape platform. On day 19, the platform was raised above the water surface (i.e., visible to the rat) as a control procedure to determine the contributions of non-spatial factors on cognitive performance. Each trial lasted until the rat climbed onto the platform or until 120 sec had elapsed, whichever occurred first. The times of the four daily trials for each rat were averaged and used in the statistical analyses.

On day 19, before the visible platform test, a single trial was provided to probe memory retention, which required removal of the platform from the pool and placing the rat in the maze at the most distal point from the target zone (i.e., quadrant where the platform was previously located) and allowing it to freely explore the pool for 30 sec. The percentage of time spent in the target zone was used in the statistical analysis. Data analyses were performed by an investigator blinded to experimental conditions, using StatView 5.0.1. Only after the analyses were concluded and the code was broken did the statistician know the composition of the groups. Motor and cognitive data were acquired over multiple days and thus were analyzed by repeated-measures analysis of variance. When the analyses of variance revealed an overall statistically significant effect, the Bonferroni^50–53^ or Newman-Keuls^49,54–59^ *post hoc* tests were utilized to determine specific group differences. Results are expressed as the mean ± standard error of the mean and were considered significant when *p* values were ≤0.05.

## Results

### Single administration studies

Kline and colleagues^50^ provided a single administration of HAL (0.5 mg/kg), the atypical APD risperidone (RISP; 0.045, 0.45, or 4.5 mg/kg), or vehicle (VEH; 1 mL/kg) 24 h after CCI injury or sham surgery. The data showed that neither HAL nor RISP negatively influenced behavioral outcome given that no statistical differences were revealed among the TBI groups receiving the different APDs relative to vehicle in motor and spatial learning performance. However, during the second phase of the study when the same APD doses were administered once-daily for 5 consecutive days, a significant reinstatement of motor and cognitive deficits was observed with both HAL and RISP. Moreover, the findings from the third phase of the study showed that the deficits in the cognitive task remained after a 3-day drug washout period in the HAL group.^50^

In contrast to the findings just described,^50^ Tang and colleagues administered HAL (0.3, 1.0, and 3.0 mg/kg; i.p.) 15 min after a mild closed head TBI produced by dropping a 21-g weight through a guide tube from a height of 25 cm onto the skull.^47^ One week later, a water-finding task that included acquisition and retention as well as a retest was utilized to assess learning and memory. The data showed that all three doses of HAL improved cognitive function relative to the VEH controls. The differential effects observed with HAL in this study versus that of Kline and colleagues^50^ may be attributable to the earlier administration of HAL in the Tang study, which may have served to attenuate TBI-induced glutamate release. The difference in behavioral tasks could also be a factor.

### Daily administration studies

Utilizing the same doses as the three-phase study,^50^ Kline and colleague (2008)^51^ showed that chronic administration (i.e., once-daily for 19 days beginning 24 h after TBI or sham injury and 1 h before behavioral assessments) of HAL and RISP significantly impeded motor and cognitive recovery, which were assessed on post-operative days 1–5 and 14–19, respectively, after CCI injury. Specifically, HAL and all three doses of RISP impaired traversal of the beam and acquisition of spatial learning relative to VEH controls. Sham controls administered the APDs were also impaired relative to VEH-treated shams. Moreover, HAL and all but the lowest dose of RISP reduced swim speed. Because of the significantly slower swimming, it was unclear whether APD-treated rats took longer to reach the platform because of treatment effect on neurotransmission or because of sedation-related swimming impairments, which would be a significant confound.^51^ Hence, to address the confound, Hoffman and colleagues (2008)^52^ provided HAL (0.5 mg/kg) and RISP (0.45 mg/kg) after the daily behavioral assessments, thus circumventing the potential sedative effect.

The data showed that only RISP delayed the return of successful beam-balance performance. HAL showed an initial deficit relative to VEH in time to traverse the beam, and both APDs showed a significant impairment in the acquisition of spatial learning relative to VEH controls, as indicated by longer path lengths and more time to find the escape platform. Importantly, administering APDs after the behavior assessments did not impact sham controls and did not alter swim speed. Quantification of cortical lesion volume and hippocampal cell survival did not reveal differences among the TBI groups, regardless of treatment.^52^ Taken together, data from both studies indicate that chronic administration of RISP and HAL impedes motor and cognitive recovery after TBI and impairs performance in uninjured controls.^51,52^

Utilizing a fluid percussion model of TBI, Wilson and colleagues (2003)^48^ compared three doses of HAL (0.03, 0.1, and 0.3 mg/kg) to saline VEH as well as three doses of the third-generation APD, olanzapine (0.3, 1.0, and 3.0 mg/kg), to VEH, which were administered beginning 24 h after a moderate injury (2.0 ± 0.1 atm) and continuing once-daily for 15 days. The data showed that chronic administration of the highest dose of HAL (0.3 mg/kg) exacerbated spatial learning deficits relative to vehicle-treated TBI rats. Olanzapine did not differ statistically from VEH in the cognitive task, and neither drug—nor doses—significantly impacted recovery of beam balance and beam walking. It is unknown whether HAL differed from olanzapine given that the drugs were not compared to one another nor to the same VEH group, which is a limitation. Although not reported, the VEH group in the olanzapine study seems to have performed worse than that of the HAL group, which may have influenced the interpretations. Regardless of insufficient group comparisons, the data showed an impairment in spatial learning after chronic HAL.^48^

Agitation after TBI is not limited to males, yet the effect of HAL in females had not been determined until Free and colleagues (2017)^49^ sought to empirically ascertain whether the deleterious effects of chronic HAL after a CCI injury of moderate severity observed in males would extend to females, and whether the outcomes would be comparable. Utilizing the paradigms of Kline and colleagues (2008)^50^ and Hoffman and colleagues (2008),^51^ anesthetized adult female and male rats received either a CCI or sham injury and then were randomly assigned to a dosing regimen of HAL (0.5 mg/kg) or VEH (1 mL/kg) that was initiated 24 h after injury and continued once-daily for 19 consecutive days. The data showed that HAL impaired the acquisition of spatial learning in female rats similarly to males, but motor was only affected in males.^49^

To understand the persistence of chronic HAL-induced detrimental effects, Phelps and colleagues (2015)^53^ randomly assigned male rats to a CCI injury or sham surgery and administered HAL (0.5 mg/kg), RISP (0.45 mg/kg), or VEH (1 mL/kg). Bromocriptine (BRO; 5 mg/kg) was also used as a positive control to determine the role of D_2_ receptor antagonism/agonism. Dosing was initiated 24 h after surgery and once-daily for 19 days. Motor and cognitive function was assessed on days 1–5 and 14–19, respectively, and again at 1 and 3 months after the withdrawal of APDs. No statistically significant differences were revealed among the groups in motor function. Acquisition of spatial learning was significantly reduced in the HAL and RISP groups during the treatment phase versus VEH and BRO. Moreover, BRO-treated rats performed significantly better than VEH. After a 1-month washout period, the TBI groups that received HAL and RISP continued to exhibit significant cognitive impairment versus BRO and VEH. By 3 months, the HAL group was still significantly impaired in the water maze, whereas the BRO group continued to improve.

These data replicated previous reports of HAL and RISP impeding cognitive recovery after TBI^50–52^ and expand the literature by revealing that deleterious effects persist for up to 3 months after drug discontinuation. The data also showed that the D_2_ receptor agonist BRO improved cognition, whereas D_2_ receptor antagonism impeded recovery after TBI.^53^

ARIP is a partial D_2_ and 5-hydroxytryptamine_1A_ receptor agonist and was selected for evaluation^54^ because other pharmacotherapies with similar receptor profiles, such as buspirone and 8-OH-DPAT, have been shown to benefit motor and cognitive outcomes after CCI injury.^60–64^ Specifically, ARIP (0.1 or 1.0 mg/kg) or VEH (1.0 mL/kg, saline VEH) were administered once-daily for 19 days beginning 24 h after a CCI or sham surgery. Motor and spatial learning and memory were assessed on days 1–5 and 14–19, respectively, and cortical lesion volume and hippocampal cell survival were quantified after the last behavior on day 19. The data revealed that the higher dose of ARIP restored beam-balance performance quicker than the lower dose and VEH. No differences were observed among the TBI groups for time to traverse the beam. In contrast, the lower dose of ARIP facilitated the acquisition of spatial learning, as indicated by reduced time and distance to the platform, and improved memory retention. Both ARIP doses reduced cortical lesion volume and spared more surviving hippocampal CA3 neurons relative to the TBI VEH control.^54^

The findings demonstrated that unlike the deleterious effects of chronic HAL,^50–52^ chronic ARIP improves neurobehavioral and cognitive outcomes and protects against histological damage after CCI injury, which suggest that it is not only a safer APD for alleviating behavioral disturbances after TBI, but also may have further rehabilitative potential by improving cognitive recovery in TBI patients.

### Intermittent administration studies

The studies reviewed thus far unequivocally demonstrate that chronic administration of HAL or RISP significantly impede the return of cognitive recovery after CCI injury in male and female rats.^50–53^ A remaining unknown is how intermittent dosing affects recovery after TBI. Knowing the outcome could be clinically relevant, given that chronic or continuous agitation may not be experienced by all patients (as opposed to context-dependent, evocable intermittent agitation), and thus daily treatment with APDs would not be warranted. To address this lingering unknown, Bao and colleagues (2019)^55^ administered HAL (0.5 mg/kg) or VEH beginning 24 h after CCI injury and continued providing it either once-daily or once every other day for 19 days to rats housed in standard (STD) or environmental enrichment (EE) conditions. EE was included to determine the effect of intermittent APD dosing on neurorehabilitation. The data replicated previous studies,^50–52^ showing that once-daily HAL after TBI blocked spatial learning relative to all other groups. In contrast, intermittent HAL did not differ from the VEH-treated, STD-housed group in spatial learning, which suggests that it did not impede acquisition. This finding, demonstrating that intermittent EE did not attenuate EE-mediated spatial learning improvements as observed with chronic HAL, indicates that HAL is not overtly detrimental when provided intermittently.^55^

Intermittent RISP dosing also lacked deleterious effects on cognitive recovery.^56^ Specifically, administering RISP (0.45 mg/kg) one, three, or seven times per week for 19 days after a CCI or sham surgery showed that only the daily RISP group exhibited motor and cognitive impairment relative to VEH controls. The groups receiving RISP 1 or 3 times per week did not differ from VEH-treated controls nor from one another.^56^

In a study designed to evaluate the effects of daily or intermittent HAL and quetiapine (QUE) on motor and cognitive recovery after TBI, Weeks and colleagues (2018)^57^ provided HAL (0.5 mg/kg) or QUE (10 mg/kg) beginning 24 h after CCI or sham surgery and continuing once-daily or once every other day for 19 days. Motor and cognitive function were assessed on days 1–5 and 14–19, respectively. The analyses indicated that daily administration of HAL impaired motor and cognitive function, as observed in previous studies.^49–53^ There were no HAL-induced impairments when provided intermittently. QUE, whether administered daily or intermittently, did not confer benefits or disrupt recovery.^57^

A potential clinical ramification of the intermittent APD studies is that HAL and RISP may be safe to manage agitation after TBI, but only when used sparingly and intermittently given that they do not appear to inhibit spontaneous recovery. In contrast, QUE does not appear to disrupt spontaneous recovery and has stronger potential for safe, continuous use.

### Daily administration studies combined with neurorehabilitation

Attempts to manage TBI-induced agitation with APDs during rehabilitation could have an impact on the process. To determine the impact of providing HAL chronically to rats undergoing EE, a rodent model of neurorehabilitation,^65–69^ Folweiler and colleagues (2017)^58^ sought to determine whether or how the two therapeutic approaches would interact and influence motor and cognitive recovery. After CCI or sham surgery, rats were provided HAL (0.5 mg/kg) or VEH (1 mL/kg) beginning 24 h after surgery and once-daily for 19 days while housed in EE or STD housing conditions. Motor and cognitive function were assessed on post-injury days 1–5 and 14–19, respectively. The data showed that EE, whether paired with HAL or VEH, recovered significantly better than the STD-housed TBI groups. Further, the TBI group administered HAL and housed in STD conditions performed worse than the STD-housed TBI group receiving VEH. The data also showed that concomitant HAL reduced the well-established therapeutic effects of EE. These findings suggest that administering HAL to TBI patients undergoing neurorehabilitation may be a double-edged sword, given that agitation must be managed but treating it with HAL may compromise therapeutic efficacy.^58^

In another study evaluating how APDs might affect neurorehabilitative outcomes, Besagar and colleagues (2019)^59^ administered ARIP (0.1 mg/kg; dose shown previously to benefit cognition^54^) or VEH beginning 24 h after CCI or sham surgery and continuing once-daily for 19 days. Rats housed in STD and EE conditions were assessed for motor and cognitive outcome as well as histopathology. ARIP, whether paired with EE or STD housing, improved beam-walk score and spatial learning and reduced cortical lesion volume relative to STD-housed VEH controls. Memory retention was only enhanced in the EE groups receiving ARIP or VEH with no between-group differences. These data indicate that ARIP enhances function and reduces histopathology after TBI, providing a more suitable alternative to APDs with D_2_ antagonist properties.^59^

## Discussion

The practice of administering APDs to manage agitation after TBI continues despite a growing literature showing that many, particularly those with D_2_ receptor antagonist properties, produce negative effects on cognitive recovery. Clinically, APDs may prolong post-traumatic amnesia and hospital/rehab length of stay, cause sedation and extrapyramidal effects, or delay motor/cognitive recovery.^34,36^ Limited case studies and case series have been performed without the ability to draw real conclusions because of a lack of adequate sample size or comparator groups.^31^ Despite the weakness of the clinical literature on the topic, robust data from pre-clinical TBI research provide critical insights into the safety and harms of APD use after TBI.

The current review, which includes all the pre-clinical studies conducted in rodents that compared behavioral outcomes in APD and VEH-treated controls, demonstrates that these drugs impair the recovery process when provided chronically, as might be the case during extended critical care or neurorehabilitation. Moreover, when chronic HAL is paired with EE, a pre-clinical model of neurorehabilitation, which on its own confers robust benefits in motor, cognitive, and histological outcomes,^65–69^ the therapeutic efficacy is significantly reduced. The clinical implication of this finding is concerning because managing agitation with HAL (and perhaps other D_2_ receptor antagonists) may compromise rehabilitation efficacy. It is noteworthy that a single administration of APDs with D_2_ receptor antagonism properties (HAL and RISP) did not markedly impact behavioral recovery relative to VEH-treated controls.

The implication of these findings is that a single administration, such as may be warranted during the initial emergency department visit after TBI, may not significantly impair subsequent motor or cognitive recovery. Intermittent HAL dosing also did not block subsequent recovery, which suggests that if a TBI patient does not display agitated or aggressive behavior every day, it may be prudent for clinicians to only prescribe as needed rather than daily. The atypical APD RISP enhances recovery after TBI and QUE has no negative effects, which make these later drugs more acceptable for treating agitated TBI patients.

Clinically, a range of treatment options are available to address post-TBI agitation, including non-pharmacological and pharmacological interventions. Recent neurocognitive rehabilitation guidelines by the French Society of Physical and Rehabilitation Medicine, International Cognitive (INCOG 2.0) and Canadian Acquired Brain Injury Knowledge Uptake Strategy (ABIKUS) emphasize non-pharmacological interventions, general minimization of medications, explicit discouragement of typical antipsychotics, and a cautious minimization approach to atypical antipsychotics.^42,70,71^ Benzodiazepines, which have differing mechanisms of action, may also be a viable alternate approach for managing agitation given that Cheng and colleagues showed that lorazepam provided for 19 days after CCI injury improved motor and cognitive outcome.^72^ An intensive care unit trial demonstrated that non-D_2_-antagonist alternative medications, such as alpha-2 agonist dexmedetomidine, outperformed HAL in reducing post-TBI agitation and delirium with lower adverse event rates.^73^

## Conclusion

The only thing that may be clear, based on clinical data at this time, is that there are no salient benefits derived from APDs and there are serious potential harms and adverse effects, both acutely and on long-term recovery. More dedicated, well-designed prospective research is needed, but early attempts have proven challenging because of the nature of the TBI patient population.^74,75^ Pre-clinical studies thus offer a highly controlled environment in which the potential benefits and harms can be systematically explored. Indeed, given the strength of data pre-clinically regarding potential harms of antipsychotic administration, recent clinical guidelines have included the citation of seminal pre-clinical works as part of the rationale to exercise caution and minimization when prescribing antipsychotic medications post-TBI.^42,70,71^

## Abbreviations Used

5-HT: serotonin
ABS: Agitated Behavior Scale
APDs: antipsychotic drugs
ARIP: aripiprazole
BRO: bromocriptine
CCI: controlled cortical impact
EE: environmental enrichment
HAL: haloperidol
i.p.: intraperitoneally
QUE: quetiapine
RISP: risperidone
STD: standard housing
TBI: traumatic brain injury
VEH: vehicle
